# AUCseg: An Automatically Unsupervised Clustering Toolbox for 3D-Segmentation of High-Grade Gliomas in Multi-Parametric MR Images

**DOI:** 10.3389/fonc.2021.679952

**Published:** 2021-06-14

**Authors:** Botao Zhao, Yan Ren, Ziqi Yu, Jinhua Yu, Tingying Peng, Xiao-Yong Zhang

**Affiliations:** ^1^ Institute of Science and Technology for Brain-inspired Intelligence, Fudan University, Shanghai, China; ^2^ Key Laboratory of Computational Neuroscience and Brain-Inspired Intelligence (Fudan University), Ministry of Education, Shanghai, China; ^3^ Department of Radiology, Huashan Hospital, Fudan University, Shanghai, China; ^4^ School of Information Science and Technology, Fudan University, Shanghai, China; ^5^ Helmholtz AI, Helmholtz zentrum Muenchen, Munich, Germany; ^6^ Ministry of Education (MOE) Frontiers Center for Brain Science, Fudan University, Shanghai, China

**Keywords:** glioma, unsupervised segmentation, MRI, toolbox, clustering

## Abstract

The segmentation of high-grade gliomas (HGG) using magnetic resonance imaging (MRI) data is clinically meaningful in neurosurgical practice, but a challenging task. Currently, most segmentation methods are supervised learning with labeled training sets. Although these methods work well in most cases, they typically require time-consuming manual labeling and pre-trained models. In this work, we propose an automatically unsupervised segmentation toolbox based on the clustering algorithm and morphological processing, named AUCseg. With our toolbox, the whole tumor was first extracted by clustering on T2-FLAIR images. Then, based on the mask acquired with whole tumor segmentation, the enhancing tumor was segmented on the post-contrast T1-weighted images (T1-CE) using clustering methods. Finally, the necrotic regions were segmented by morphological processing or clustering on T2-weighted images. Compared with K-means, Mini-batch K-means, and Fuzzy C Means (FCM), the Gaussian Mixture Model (GMM) clustering performs the best in our toolbox. We did a multi-sided evaluation of our toolbox in the BraTS2018 dataset and demonstrated that the whole tumor, tumor core, and enhancing tumor can be automatically segmented using default hyper-parameters with Dice score 0.8209, 0.7087, and 0.7254, respectively. The computing time of our toolbox for each case is around 22 seconds, which is at least 3 times faster than other state-of-the-art unsupervised methods. In addition, our toolbox has an option to perform semi-automatic segmentation *via* manually setup hyper-parameters, which could improve the segmentation performance. Our toolbox, AUCseg, is publicly available on Github. (https://github.com/Haifengtao/AUCseg).

## Introduction

High-grade gliomas (HGG) are the most common type of central nervous cancer among adults. It has the characteristics of rapid growth, blurred margins, irregular shapes, and invading into the surrounding tissue (1). Currently, HGG segmentation in magnetic resonance imaging (MRI) plays an important role in clinical treatment (2). Manually labeling gliomas in MRI images by doctors has been regarded as the gold standard of tumor segmentation. But it is a tedious and time-consuming job. Several studies have reported that the variabilities of manual tumor segmentation are over 20% (2–4). In the past two decades, computer-aided methods for the segmentation of HGG have been used to save time for clinicians and address the problem of manual variabilities. Despite the emergence of many excellent algorithms in recent years, the segmentation of HGG is still a challenging job (5).

Deep learning-based methods have achieved high Dice similarity in HGG segmentation. The U-net (6) based network architectures were widely used in this task and performed well. Myronenko et al. (7) proposed a 3D U-net with autoencoder regularization, which ranked the top-1 in BraTS2018. Later, Jiang et al. (8) using the two-stage cascaded U-net won the first prize in BraTS2019. Recently, T Henry et al. (9) took first place in BraTS2020 by a deep supervised 3D U-net. Although these supervised methods perform well for HGG segmentation, they require a large amount of labeled data. However, labeling tumors manually not only requires medical expertise, but also is a time-consuming task.

By contrast, clustering is an unsupervised method, which does not require labeling data for training. Vijay J et al. proposed an HGG segmentation method based on K-means clustering, which can quickly segment the whole tumor from 2D images (10). But simply clustering could cause inaccurate results because of the image noise. To solve this, Tripathy et al. (11) improved the fuzzy c-means with spatial context information and intuitionistic set, also named SIFCM. However, this method is time-consuming. Cai et al. proposed the FGFCM algorithm, which reduced the computing time by only considering the partial value instead of the whole image size (12). However, the above advanced clustering methods have not been applied to HGG segmentation because of the lack of a suitable pipeline.

In addition to clustering-based methods, other unsupervised segmentation methods have been investigated for HGG segmentation. Guo et al. (13) reported a semi-automatic method for the segmentation of HGG based on active contour, which was evaluated on 20 cases (a small portion of BraTS2013 training data) and could segment the whole tumor (WT), the tumor core (TC), necrotic (NC) and enhancing tumor (ET). However, this method requires a region of interest (ROI) provided by the user. Juan-Albarracín et al. (14) further proposed an automatic strategy for the HGG segmentation based on Gaussian Hidden Markov Random Field (GHMRF) on the 21 cases from BraTS2013. However, this method is slow and takes 140 ± 32 minutes for the whole segmentation pipeline. N. Sauwen et al. (15) proposed a method based on hierarchical non-negative matrix factorization (HNMF) and gained acceptable segmentation performance on two independent cross-site datasets (21 cases and 14 cases, respectively).

Although the above unsupervised methods achieved acceptable performance, they used a small dataset with limited sample size so that the conclusions may result in bias. To provide clinicians with more robust and reliable assistance, a much larger dataset should be used to have a more thorough evaluation. Moreover, a ready-to-use toolbox is a big advance for its clinical translation. To address these issues, we aim to propose an automatically unsupervised tumor segmentation strategy based on clustering and morphological methods, and to evaluate our method on BraTS2018, a much larger dataset that includes over 200 cases (2–4). Furthermore, we release our segmentation pipeline as a toolbox to provide clinicians with assistance.

## Methods

### Pipeline

**Figure 1:**
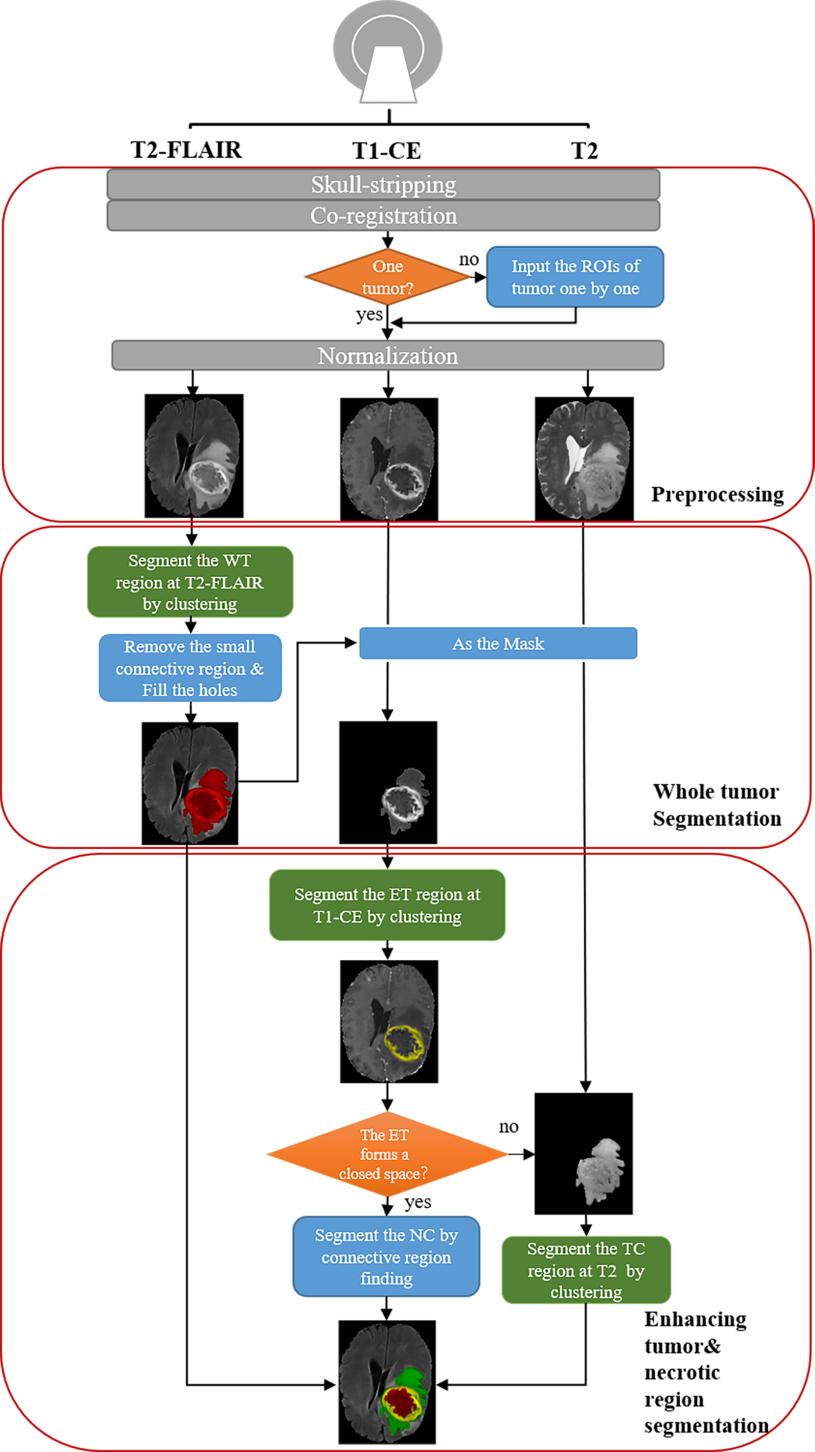
Schematic diagram of our automatic unsupervised pipeline. The orange and green boxes indicate a hyper-parameter there.

The pipeline is shown in **Figure 1**. There are three steps for preprocessing: 1) skull-stripping; 2) co-registration on different MRI modalities; 3) Normalization (0~1).

For segmentation, the core concept of this pipeline is based on the pathological and radiological characteristics of HGG. In the T2-FLAIR images, the HGG edema region shows higher signal intensity than other regions due to high water content. In post-contrast T1-weighted images (T1-CE) images, enhancing area shows a significantly higher signal intensity, which means the damage to the blood-brain barrier. Based on the above two features, the edema and enhancing area of the tumor can be first segmented from T2-FLAIR images and T1-CE images by clustering and selecting the subclass with the highest intensity. According to the characteristics of tumor growth, edema is generally located on the boundary of the tumor, while the necrotic (NC) area is generally within the tumor. Therefore, we can fill the connected domain inside the edema region by analyzing the connected components and regard the edema region as the part of the whole tumor (WT) region. After that, the WT was used as the ROI on T1-CE images and segment the enhancing tumor (ET) on it using clustering. The tumor core (TC) is composed of NC and ET.

Additionally, because NC often occurs in the interior of the rapidly growing area of the tumor (the enhancing area), the necrotic area can be segmented using the connected component analysis. However, due to image quality and clustering accuracy, the segmented enhancing regions may not constitute a closed connected domain. When there is little or no enhancing regions, our pipeline still performs well for the segmentation of WT and TC. Note that for these cases the NC cannot be segmented directly due to lacking a completely connected domain. To solve the problem, we propose an alternative solution that segments the tumor core by clustering intensities in the T2 images instead of T2-FLAIR images (**Supplementary Figure S1**). Therefore, the NC region can be segmented by subtracting ET from TC. With above concept, the WT, the TC, the ET, and the NC can be successfully segmented in most cases.

Moreover, we also provide an option to segment two or more tumors (**Supplementary Figure S2**). When there is more than one tumor, the hyper-parameter ‘ROI’ (**Table 1**) is provided for tumor segmentation. Then tumors can be segmented using our pipeline one by one.

**Table 1:** Hyper-parameters in our pipeline.

Hyper-params	Default	Describe
n_cluster1	5	Number of clustering for WT segmentation.
n_cluster2	3	Number of clustering for ET segmentation.
ROI	None	If you provide a ROI, the raw image will be cropped by it. If more than one tumor present, it is required to provide ROIs of each tumor for the segmentation.
nc_seg_mode	cc	We provide two modes to segment the necrotic region, ‘cc’ and ‘t2’. If the ET region could not wrap up the NC area, it would be better to choose mode ‘t2’. When you choose ‘t2’, the T2 image must be provided.
n_cluster3	3	Number of clustering for TC segmentation. If the “cc” is chosen as the ‘nc_seg_mode’, n_cluster3 will not be used.

Based on the proposed pipeline, our method has the five hyper-parameters as shown in **Table 1**.

### Clustering Algorithms

#### K-Means and Mini-Batch K-Means

K-Means clustering is an unsupervised unstructured iterative partitioning method based on distance. K-means builds a distance model:

(1) $$J=\Sigma_{j=1}^{k}\Sigma_{i=1}^{n}||x_{i}-c_{j}|{|}^{2}$$

In (1), k represents the number of classes; N is the number of elements to be clustered; ||x_i_-c_j_||^2^ is the distance between points x_i_ and c_j_, usually using Euclidean distance. The process of clustering is to find the parameter that minimizes J. Mini-batch K-means (16) is an optimization for K-means clustering. The main idea of this method is that mini-batches have lower stochastic noise than classic stochastic gradient descent, but do not suffer the large computational cost.

#### Fuzzy C Means (FCM)

FCM introduces the concept of membership degree in fuzzy mathematics based on k-means clustering. After introducing a membership degree, the distance model becomes as follows:

(2) $$\left\{u_{ij}\right\}=arg\;min\;\Sigma_{j=1}^{c}\Sigma_{i=1}^{n}u_{ij}^{m}|\left|x_{i}-c_{j}\right|{|}^{2},\mathrm{s.}\mathrm{t.}\;\Sigma_{j=1}^{c}u_{ij}=1\left(i=1,2,3,\ldots ,n\right)$$

FCM algorithm is used for clustering, which is to solve the minimum value of equation (2). The $u_{ij}^{m}$ is the membership of element i for class j. It is a conditional extremum problem. The local extremum can be obtained by using the Lagrange multiplier method to incorporate the constraint conditions into the model.

#### Gaussian Mixture Model (GMM)

GMM uses a probability model to express the clustering prototype. Multidimensional Gaussian distribution is hypothesized for each class. The Gaussian mixture distribution is as:

(3) $$p(x)=\frac{1}{{\left(2\pi \right)}^{\frac{n}{2}}{\left|\text{Σ}\right|}^{\frac{1}{2}}}e^{-\frac{1}{2}{\left(x-\mu \right)}^{\text{T}}{\text{Σ}}^{-1}(x-\mu )}$$

(4) $$p_{M}(x)=\Sigma_{j=1}^{c}\alpha_{j}\cdot p\left(x|\mu_{j},\Sigma_{j}\right)\;,\;\;\Sigma_{j}^{c}\alpha_{j}\;=\;1$$

Where formula (3) is the multi-dimensional Gaussian distribution, Ʃ is the covariance matrix, *µ* is the mean vector. Formula (4) represents the Gaussian mixture distribution. Where *α_j_* represents the mixture coefficient and the probability of the Jth Gaussian distribution. The maximum likelihood method is used to solve the parameters of equation (5):

(5) $$\{\alpha_{j},\mu_{j},\Sigma_{j}\}=arg\;max_{\alpha ,\mu ,\;\;\Sigma }\ln\left(\Pi_{i}^{n}p_{M}(x_{i})\right)$$

Since equation (5) contains hidden variables, the Expectation-Maximization (EM) algorithm is generally used to optimize parameters. After the Gaussian distribution is known, we divide the elements according to the posterior probability corresponding to the prototype, that is:

(6) $$\lambda_{i}=\arg\;\max_{j\in \{1,2,\text{...},k\}}\frac{\alpha_{j}\cdot p\left(x_{i}|\mu_{j},\Sigma_{j}\right)}{\Sigma_{l=1}^{k}\alpha_{l}\cdot p\left(x_{i}|\mu_{l},\Sigma_{l}\right)}$$

### Morphological Methods

The morphological methods were used for dilation and connected components analysis. The connected component analysis is to find the aggregation region of the same voxel. There are three levels of connectivity for 3D images. The analysis of connected components adopts the accelerated algorithm proposed by Wu et al. (17), which greatly reduces the computing time. Dilation refers to local maximum substitution, which is to calculate the maximum value of pixels in the region covered by the core to replace centrosomes, as shown in equation (7).

(7) $$dst_{dilate}(x,y)=\underset{(x^{\prime },y^{\prime })\in kernel}{\max}src(x^{\prime },y^{\prime })$$

### Evaluation

Multi-parametric MRI images of 210 HGG patients from BraTS2018 (2–4) training sets were used to evaluate the pipeline. Our method was implemented with Python3.6, and the main external packages included Numpy, Scikit-learn, Scikit-image, and so on. The experiments were run at the workstation DELL FC430 with CentOS 7.5.1804, Intel (R) Xeon(R) E5-2640 v4 2.4GHz and the memory 256GB. To search for the most suitable clustering method, the main hyper-parameters of the model were set as the default value shown in **Table 1**. K-means++ method or K-means is adopted in the clustering to initialize the clustering center. Dice coefficients, false-positive volume fractions, and false-negative volume fractions were calculated to evaluate the segmentation results. We also adjusted the hyper-parameters to evaluate the performance of our pipeline and analyzed the hyper-parameters distribution. Because the hyper-parameter ‘roi’ is subjective, we mainly adjusted the remaining four hyper-parameters.

## Results

### Comparison of Clustering Algorithms

Using the same default hyper-parameters, we compared tumor segmentation results with different clustering methods. It can be found that K-means is faster than other methods (**Table 2**).

**Table 2:** Computing time per patient (second).

	K-means	Mini-batch K-means	GMM	FCM
Time	**10.28 ± 1.95**	14.49 ± 3.64	19.12 ± 4.27	181.20 ± 51.87

The value in bold means the best performance.

The DICE coefficients of segmentation with different clustering methods are shown in **Table 3**. The dice coefficient lower than 0.5 is considered as failed detection. The highest mean values of DICE coefficients and success rates are shown in bold. It can be seen that GMM has the best performance in our pipeline.

**Table 3:** Dice index of different clustering methods for WT, TC, and ET.

		WT	TC	ET
K-means	Mean ± Std	0.8248 ± 0.1092	0.6975 ± 0.1162	**0.7266 ± 0.0975**
	Success Case	155/210	104/210	135/210
Mini-batch K-means	Mean ± Std	0.8147 ± 0.1093	0.6833 ± 0.1249	0.7168 ± 0.1019
	Success Case	151/210	99/210	127/210
GMM	Mean ± Std	0.8209 ± 0.1051	**0.7087 ± 0.1210**	0.7254 ± 0.1080
	Success Case	**161/210**	**120/210**	**149/210**
FCM	Mean ± Std	**0.8293 ± 0.1045**	0.6874 ± 0.1158	0.7218 ± 0.0988
	Success Case	147/210	100/210	126/210

The value in bold means the best performance.

**Table 4** shows the DICE, false positive, and false negative of segmentation results with GMM clustering in this pipeline. The whole tumor segmentation under this parameter has a problem of over-segmentation, while the segmentation of the tumor core and enhancing region has a problem of under-segmentation. One possible reason for the problem is that we use the same default parameters for different cases.

**Table 4:** Results of the GMM based HGG segmentation pipeline.

	WT	TC	ET
DICE	0.8209 ± 0.1051	0.7087 ± 0.1210	0.7254 ± 0.1080
FPVN	0.2600 ± 0.3049	0.1228 ± 0.1714	0.2030 ± 0.3042
FNVN	0.1325 ± 0.1438	0.3732 ± 0.1648	0.3163 ± 0.162

**Figure 2:**
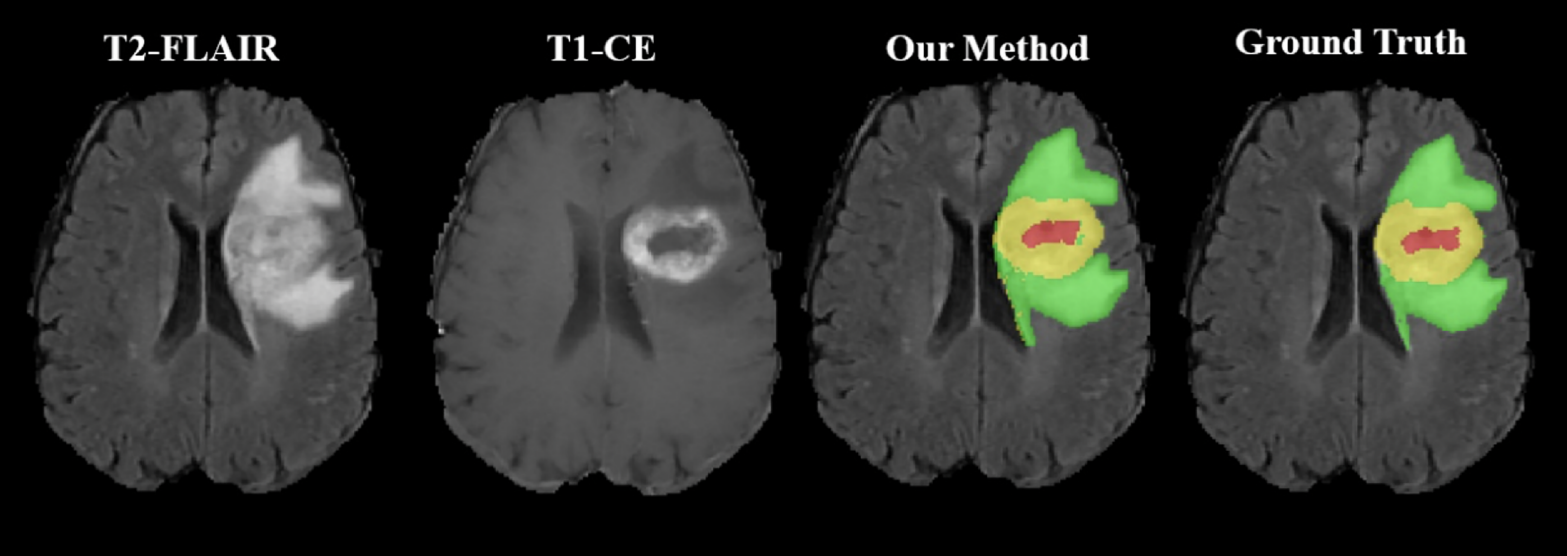
Representative segmentation results using our toolbox. The green area represents the tumor edema area; the yellow area represents the ET area; the red area represents the NC area.

As shown in **Figure 2**, the WT, the TC, and the ET area can be isolated using our method. TC is composed of NC and ET areas.

### Adjusting Hyper-Parameters

**Table 5** shows the segmentation result after adjusting the hyper-parameters, including ‘n_cluster1’, ‘n_cluster2’, ‘nc_seg_mode’, and ‘n_cluster3’. Compared with the results before adjusting shown in **Table 4**, there is a great improvement in Dice and Success Case rate.

**Table 5:** Comparison of segmentation results with manually adjusted hyper-parameters.

	WT	TC	ET
DICE	0.8420 ± 0.0982	0.7531 ± 0.1093	0.7496 ± 0.1005
FPVN	0.1836 ± 0.2091	0.1287 ± 0.1476	0.1436 ± 0.1884
FNVN	0.1438 ± 0.1180	0.3123 ± 0.1425	0.3095 ± 0.1458
Success Case	198/210	171/210	180/210

**Figure 3:**
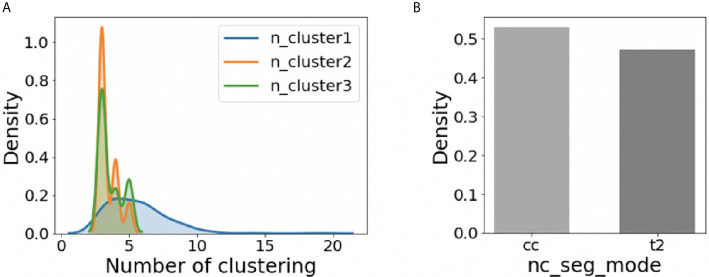
The distribution of hyper-parameters on BraTS2018 HGG training data (n=210). **(A)** The distribution of ‘n_cluster1’, ‘n_cluster2’, and ‘n_cluster3’. **(B)** The distribution of ‘nc_seg_mode’.

For the whole 210 cases, the distribution of adjusted hyper-parameters ‘n_cluster1’, ‘n_cluster2’, and ‘n_cluster3’ was plotted in **Figure 3A** using the kernel density estimation method. The distribution of ‘nc_seg_mode’ was shown in **Figure 3B**. We could find that the suitable ‘n_cluster1’ is mainly in the range of 3~10, and both ‘n_cluster2’ and ‘n_cluster3’ were mainly in the range of 3~5. As for the ‘nc_seg_mode’, ‘cc’ and ‘t2’ were equally likely to be chosen. So, there are mainly 96 (8*3*4) choices, because the ‘n_cluster3’ would not be used if ‘cc’ was chosen. In practice, we do not have to try every possible option and adjust the parameters in the order of ‘n_cluster1’, ‘n_cluster2’, ‘nc_seg_mode’, and ‘n_cluster3’. For clustering, the large number of subclasses always means big FN. We can use this to speed up the tuning of parameters.

### Comparison With Other Unsupervised Methods

As shown in **Table 6**, we compare our methods with several state-of-the-art unsupervised methods. With our method, the WT, TC, and ET can be automatically segmented using default hyper-parameters with Dice score 0.8209, 0.7087, and 0.7254, respectively, which are similar to the published methods. Note that we tested our methods in 210 cases from BraTS2018, which is almost 10 times larger than the datasets other methods used. Another highlight is that the average computing time of our toolbox for each case using the default hyper-parameters is 19 seconds. If adjusted hyper-parameters are used, the average computing time for the segmentation of each case is 22 seconds, which is still comparable to the default setting. As shown in **Table 7**, both settings are at least 3 times faster than other unsupervised methods.

**Table 6:** Performance comparison of several unsupervised methods.

Author	Method	Testing data	Cases	WT	TC	ET
Guo et al. (13)	Active contour	BraTS2013	20	0.82	0.82	0.71
Juan-Albarracín et al. (14)	GHMRF	BraTS2013	21	0.72	0.62	0.59
N. Sauwen et al. (15)	HNMF	UZ Gent	21	0.81	0.74	0.68
		UZ Leuven	14	0.85	0.84	0.73
Our method (Default hyperparameters)	GMM	BraTS2018	210	0.82	0.71	0.73
Our method (Adjusted hyperparameters)	GMM	BraTS2018	210	0.84	0.75	0.75

**Table 7:** Comparison of computing time (seconds).

Methods	Juan-Albarracín et al. (14)	Guo et al. (13)	N. Sauwen et al. (15)	Our method (Default hyperparameters)	Our method (Adjusted hyperparameters)
Average Time	7620	60	208	19	22

## Discussion

We propose an automatically unsupervised clustering method for 3D-segmentation of HGG on multi-parametric MR images. Compared with previous unsupervised methods, our method achieved a stable performance in BraTS2018 dataset including 210 subjects, which is much larger than the previous studies. On the other hand, our method takes less computing time (**Table 7**). Therefore, it may have a broad application, such as clinical translation, preprocessing for supervised learning, etc.

### Comparison of Unsupervised Learning Methods

Among the methods of unsupervised learning, the method-based Markov random field and gaussian hybrid model (GHMRF) ranked first among the same kind of unsupervised methods in BraTS2013 challenge (14). Supervised methods based on deep learning have been used in the BraTS challenge since 2013. The two-stage Cas-Cascade U-Net method proposed by Jiang et al. won the championship in BraTS2019 (8). In addition, N. Sauwen et al. (15) introduced more types of MRI data for HGG segmentation, including magnetic resonance spectroscopic imaging (MRSI) et al. They proposed a method based on hierarchical non-negative matrix factorization (HNMF), and tested it on two independent cross-site datasets, which reached a higher place among unsupervised HGG segmentation methods. Compared with the above methods, our method performs well among unsupervised methods although the performance of our method is not as good as supervised method (**Table 6**). According to BraTS2018 Leaderboard (https://www.cbica.upenn.edu/BraTS18/lboardValidation.html), the Dice for ET, WT, and TC are in range of 0.517~0.825, 0.618~0.913, and 0.537~0.872. Note that the performance of our method is in the similar range with most of deep leaning-based methods for the segmentation of brain tumors. Although some state-of-the-art deep leaning-based models have achieved higher performance than our method, their interpretability remains unclear. By contrast, the clustering model we used is interpretable and maybe easier for the clinical translation, which is another advantage of our method.

### Limitations

Although adjusting hyper-parameters can improve the segmentation performance with default hyper-parameters, our method does not perform well for a few cases. We have summarized potential causes for suboptimal segmentation performance with low segmentation DICE scores, which can be roughly grouped into four categories (**Supplementary Figure S3**): a) uncompleted skull stripping; b) abnormal white matter hyperintensities caused by pathological change, such as demyelination; c) low contrast difference between tumor and normal tissue, and d) blurred images caused by head motion or other reasons.

## Conclusion

We proposed a novel 3D-unsupervised method and implemented a toolbox based on that to automatically segment the whole HGG, tumor core, and enhancing tumor in MR images with Dice score 0.8209, 0.7087, and 0.7254, respectively using default hyper-parameters. Our toolbox has the option to do semi-automatic segmentation *via* manually adjusting hyper-parameters, which could further improve segmentation performance. The combination of GMM with our method performs better than K-means, Mini-Batch K-means, and Fuzzy C Means (FCM). Besides, the computing speed of our method is faster than other unsupervised pipelines. We release our toolbox to provide clinicians with assistance.

## Data Availability Statement

The original contributions presented in the study are included in the article/**Supplementary Material**. Further inquiries can be directed to the corresponding author.

## Author Contributions

BZ: conceived this study, analyzed the data, and drafted the initial manuscript. YR: discussed and finalized the manuscript. ZY: analyzed the data, discussed and finalized the manuscript. JY: discussed and finalized the manuscript. TP: discussed and finalized the manuscript. X-YZ: conceived this study, interpreted the results, and wrote the manuscript. All authors contributed to the article and approved the submitted version.

## Funding

This study was supported in part by grants from the Shanghai Science and Technology Committee (20ZR1407800), the National Natural Science Foundation of China (81873893), Shanghai Municipal Science and Technology Major Project (No.2018SHZDZX01), ZJ Lab, and Shanghai Center for Brain Science and Brain-Inspired Technology, Fudan University original project (IDH2306024/007), and Shanghai Science and Technology Commission (18411967300).

## Conflict of Interest

The authors declare that the research was conducted in the absence of any commercial or financial relationships that could be construed as a potential conflict of interest.
