# Supplementary material for: AUCseg: An Automatically Unsupervised Clustering Toolbox for 3D-Segmentation of High-Grade Gliomas in Multi-Parametric MR Images
Source: Front Oncol. 2021 Jun 14;11:679952. doi: 10.3389/fonc.2021.679952 (PMC8236895; doi:10.3389/fonc.2021.679952)
Supplement: Supplementary file 1 [file DataSheet_1.pdf]

Supplementary results:

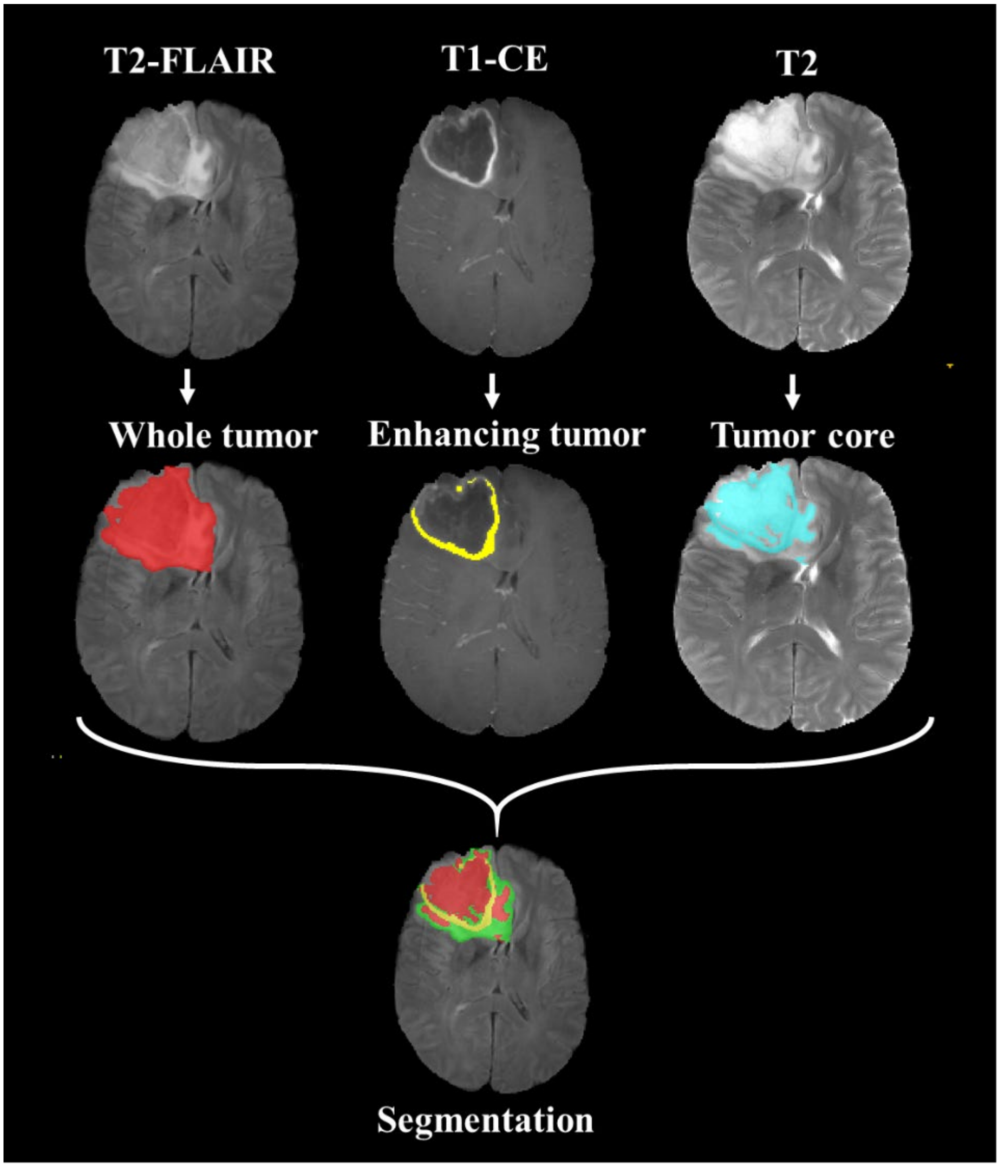

Figure S1. Examples of tumor segmentation in the condition of little enhancing region (ET) within tumor. The hyper-parameter ‘nc_seg_mode’ is set as ‘T2’ for segmentation when the ET region cannot wrap the NC area. The tumor core can be segmented by clustering in the T2 images instead of T2-FLAIR images.

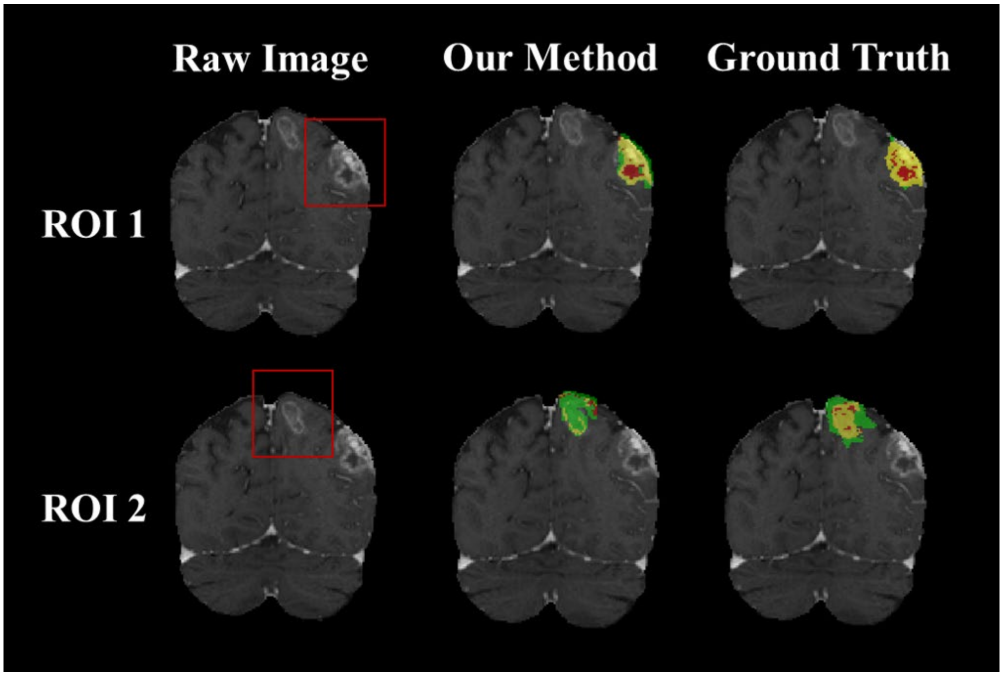

Figure S2. Examples of two tumor segmentation. First, the hyper-parameter ‘ROI’ should be provided for segmentation when there is more than one tumor region. Then, tumors can be segmented one by one using our pipeline. The red boxes indicate ROIs.

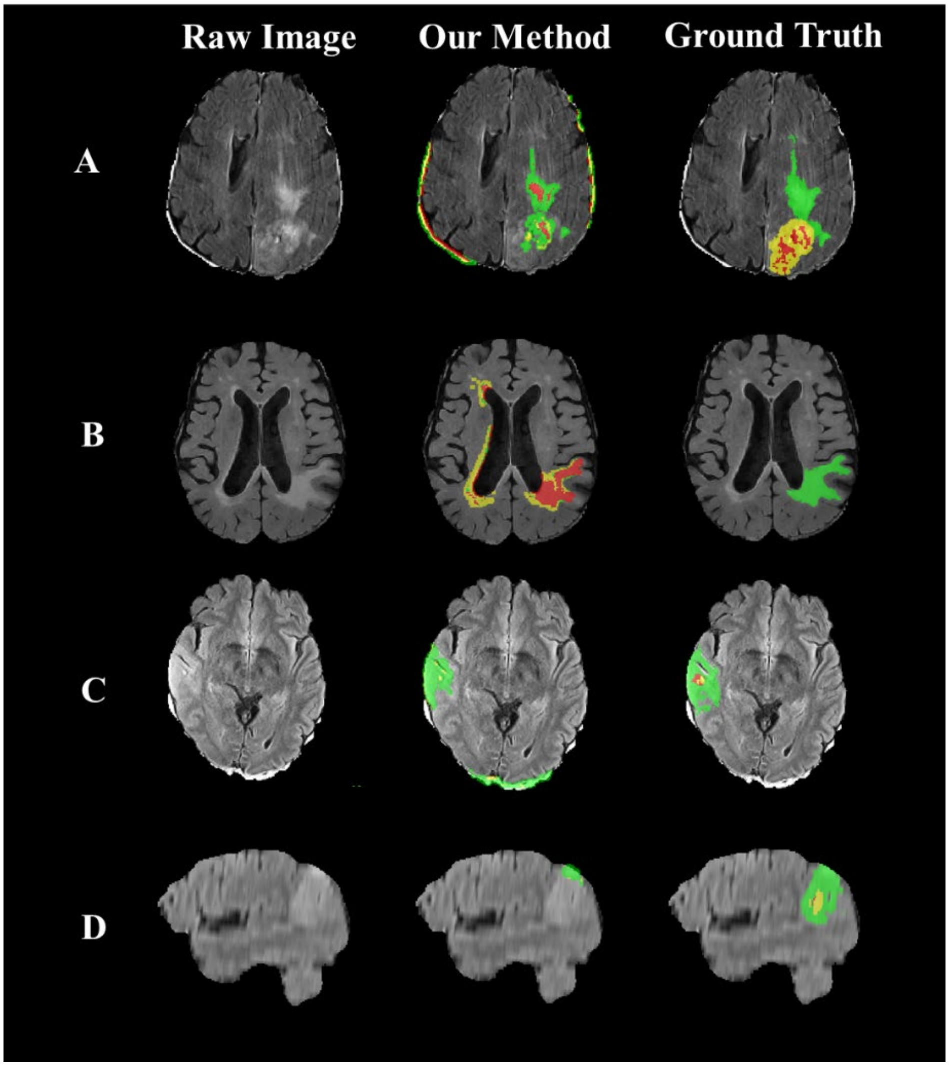

Figure S3. Examples of segmentation results with low DICE scores. There are grouped into four categories: A) Uncompleted skull stripping; B) Abnormal white matter hyperintensities caused by pathological change, such as demyelination; C) Low contrast difference between tumor and normal tissue; D) Blurred images caused by head motion or other reasons.
